# Predictors of Implant Subsidence and Its Impact on Cervical Alignment Following Anterior Cervical Discectomy and Fusion: A Retrospective Study

**DOI:** 10.3390/jcm14165660

**Published:** 2025-08-10

**Authors:** Rose Fluss, Alireza Karandish, Rebecca Della Croce, Sertac Kirnaz, Vanessa Ruiz, Rafael De La Garza Ramos, Saikiran G. Murthy, Reza Yassari, Yaroslav Gelfand

**Affiliations:** Leo M. Davidoff Department of Neurological Surgery, Montefiore Medical Center, Albert Einstein College of Medicine, Bronx, NY 10467, USA

**Keywords:** anterior cervical discectomy and fusion, subsidence, spinal alignment, standalone implants, cervical lordosis

## Abstract

**Background/Objectives**: Anterior cervical discectomy and fusion (ACDF) is a common procedure for treating cervical spondylotic myelopathy. Limited research exists on the predictors of subsidence following ACDF. Subsidence can compromise surgical outcomes, alter alignment, and predispose patients to further complications, making it essential to prevent and understand it. This study aims to identify key risk factors for clinically significant subsidence and evaluate its impact on cervical alignment parameters in a large, diverse patient population. **Methods:** We conducted a retrospective review of patients who underwent ACDF between 2013 and 2022 at a single institution. Subsidence was calculated as the mean change in anterior and posterior disc height, with clinically significant subsidence being defined as three millimeters or more. Univariate analysis was followed by regression modeling to identify subsidence predictors and analyze patterns. Subgroup analyses stratified patients by implant type, number of levels fused, and cage material. **Results**: A total of 96 patients with 141 levels of ACDF met the inclusion criteria. Patients with significant subsidence were younger on average (52.44 vs. 55.94 years; *p* = 0.074). Those with less postoperative lordosis were more likely to experience significant subsidence (79.5% vs. 90.2%; *p* = 0.088). Patients with significant subsidence were more likely to have standalone implants (38.5% vs. 16.7%; *p* < 0.01), taller cages (6.62 mm vs. 6.18 mm; *p* < 0.05), and greater loss of segmental lordosis (7.33 degrees vs. 3.31 degrees; *p* < 0.01). Multivariate analysis confirmed that standalone implants were a significant independent predictor of subsidence (OR 2.679; *p* < 0.05), and greater subsidence was positively associated with loss of segmental lordosis (OR 1.089; *p* < 0.01). Subgroup analysis revealed that multi-level procedures had a higher incidence of subsidence (35.7% vs. 28.1%; *p* = 0.156), and PEEK cages demonstrated similar subsidence rates compared to titanium constructs (28.1% vs. 29.4%; *p* = 0.897). **Conclusions**: Standalone implants are the strongest independent predictor of significant subsidence, and those that experience subsidence also show greater loss of segmental lordosis, although not overall lordosis. These findings have implications for surgical planning, particularly in patients with borderline bone quality or requiring multi-level fusions. The results support the use of plated constructs in high-risk patients and emphasize the importance of individualized surgical planning based on patient-specific factors. Further research is needed to explore these findings and determine how they can be applied to improve ACDF outcomes.

## 1. Introduction

Cervical spondylotic myelopathy is the leading cause of spinal cord dysfunction worldwide, often presenting with symptoms such as paresthesias, hand clumsiness, weakness, and significant imbalance, which can severely disrupt a patient’s quality of life [1]. The pathophysiology of cervical spondylotic myelopathy involves a complex interplay of static and dynamic factors that result in spinal cord compression and dysfunction. Static factors include disc degeneration, osteophyte formation, ligamentum flavum hypertrophy, and ossification of the posterior longitudinal ligament. Dynamic factors encompass repetitive trauma during neck motion, particularly in the setting of developmental or acquired spinal canal stenosis. Anterior cervical discectomy and fusion (ACDF) is a widely used surgical procedure, first developed in 1958, aimed at halting the progression of these symptoms [2,3]. The procedure was pioneered by Robinson and Smith, who described the technique of anterior cervical discectomy followed by interbody fusion. Over the decades, ACDF has evolved significantly, with improvements in surgical technique, implant design, and understanding of cervical biomechanics. The procedure has become the gold standard for treating single- and multi-level cervical degenerative disease with radiculopathy or myelopathy. ACDF involves an anterior approach to remove the damaged intervertebral disc to be replaced with a cage, typically filled with either allograft or autologous bone. The surgical approach provides excellent visualization of the neural elements while avoiding the morbidity associated with posterior approaches. The anterior approach allows for direct decompression of the neural elements by removing the offending disc material and osteophytes, while simultaneously providing the opportunity for interbody fusion to restore disc height and maintain cervical lordosis. Cages are commonly made from materials such as polyetheretherketone (PEEK), titanium, or bone allograft. Key factors in the choice of implants include its height, material, and whether a rigid plate can be used for fixation versus using a standalone implant [4]. PEEK cages have gained popularity due to their radiolucency, which facilitates assessment of fusion, and their elastic modulus, which closely approximates that of bone. Titanium cages offer superior strength and biocompatibility but may obscure radiographic assessment of fusion. The decision between standalone and plated constructs represents a critical choice in ACDF surgery. Plated constructs provide additional stability through rigid fixation, which can reduce micromotion and potentially improve fusion rates. However, they require additional surgical time, may increase the risk of dysphagia, and can lead to stress shielding. Standalone cages, while simpler to implant and associated with reduced operative time, rely solely on the inherent stability of the cage design and may be more susceptible to subsidence. Postoperatively, a known phenomenon is the subsidence of the implant into the adjacent vertebrae [5]. While some degree of subsidence is expected, research indicates that excessive subsidence can negatively affect spinal alignment and, in severe cases, may exacerbate radiculopathy symptoms, potentially requiring reoperation [6,7]. The biomechanical factors contributing to subsidence are multifactorial and include endplate preparation, cage design, bone quality, and postoperative loading. Excessive endplate preparation can weaken the subchondral bone, while inadequate preparation may impair fusion. The cage design, including its footprint, surface texture, and height, influences the contact area and stress distribution on the endplates. Bone quality plays a crucial role in subsidence, with osteoporotic patients being at higher risk due to decreased bone mineral density and compromised trabecular architecture. Patient factors such as age, smoking status, and nutritional status can significantly impact bone health and healing potential. Understanding these factors is essential for optimal surgical planning and patient counseling. Despite technological advancements and increased understanding of ACDF biomechanics, clinical outcomes related to subsidence remain variable. This variability is compounded by differences in implant design, patient anatomy, surgical technique, and bone quality. Although numerous studies on subsidence following ACDF exist, few have focused on identifying independent risk factors for its occurrence or its effect on post-op alignment. Many of these studies suffer from small sample sizes, inconsistent findings, or are conducted in patient populations that differ from those at our institution [8,9,10,11]. Furthermore, studies often use differing criteria to define subsidence, which complicates cross-study comparisons and limits the ability to develop universal recommendations. The definition of clinically significant subsidence varies considerably in the literature, ranging from 1 mm to 5 mm. This variability makes it challenging to compare studies and establish evidence-based guidelines. Some authors argue that any measurable subsidence is clinically relevant, while others contend that only subsidence exceeding 3 mm is associated with clinical symptoms or radiographic changes.

The goal of this study is to identify risk factors for subsidence and further explore its impact on alignment parameters using a comprehensive and diverse patient database. By clarifying these associations, we aim to guide clinical decision-making, implant selection, and preoperative patient counseling, ultimately improving surgical planning and outcomes. Additionally, this study seeks to provide insights into the relationship between subsidence and segmental versus global cervical alignment, which may have implications for adjacent segment disease and long-term clinical outcomes.

## 2. Materials and Methods

### 2.1. Data Collection

Patients who underwent ACDF between 2013 and 2022 at a single large urban institution were identified for inclusion in this study. Elective cases were selected, provided that they had follow-up radiographic imaging available within two weeks and at least three months postoperatively. The minimum follow-up period of three months was chosen to allow for adequate healing and stabilization of the construct, while ensuring that early postoperative changes could be distinguished from true subsidence. Exclusion criteria included the absence of necessary imaging, patients under 18 years of age, those with trauma or oncology-related conditions, and patients who had previously undergone cervical spine surgery. To improve data reliability and reduce confounding, patients with significant preoperative deformity or prior cervical fusion were also excluded. Patients with inflammatory arthropathies, such as rheumatoid arthritis or ankylosing spondylitis, were also excluded due to their potential impact on bone quality and fusion outcomes. Additionally, patients with active infections or those undergoing revision surgery were excluded to minimize confounding variables. All procedures were performed by board-certified spine surgeons using standardized operative techniques. The surgical team consisted of four fellowship-trained spine surgeons with at least 5 years of experience in cervical spine surgery. Standardized protocols were followed for patient positioning, approach, decompression, and implant placement to minimize technical variability. The following variables were collected for each patient: age, sex, hemoglobin levels, number of levels fused/operated, cage material, and cage height. Additionally, surgical details such as the number of levels treated, standalone versus plated constructs, and intraoperative factors, including surgery duration, were recorded. This study was approved by the Institutional Review Board (IRB) of The Albert Einstein College of Medicine (IRB No. 2016-6896, approval date: 21 April 2021, reapproval date: 23 July 2025). Patient consent was waived due to the retrospective nature of the study and the use of de-identified data. All data were collected and stored in compliance with HIPAA regulations and institutional privacy policies.

### 2.2. Radiographic Measurements

The anterior and posterior disc heights, cervical lordosis, segmental lordosis, and sagittal vertical axis (SVA) were measured both in the immediate postoperative period and at the last follow-up. Radiographic measurements were performed using standardized techniques on lateral cervical spine radiographs obtained in the neutral position. Disc height measurements were performed with anterior disc height being measured as the distance between the anterior–superior endplate of the inferior vertebra and the anterior–inferior endplate of the superior vertebra. Posterior disc height was measured similarly using the posterior endplates. Care was taken to ensure consistent measurement points and to account for any magnification differences between radiographs. Subsidence was calculated as the mean difference in the anterior and posterior disc heights from the immediate postoperative scan to the last follow-up. Clinically significant subsidence was defined as a change of ≥3 mm. This threshold was chosen based on previous research suggesting that subsidence of 3 mm or greater is associated with clinical symptoms and radiographic changes. The mean of anterior and posterior measurements was used to account for asymmetric settling that may occur with subsidence. Changes in SVA, cervical lordosis, and segmental lordosis were also assessed, with a positive change in angle indicating a decrease in lordosis. Plain radiographs of the cervical spine were used to measure cervical alignment characteristics. Ultimately, two patient groups were created: one group with more than 3 mm of subsidence and another group without significant subsidence.

### 2.3. Statistical Analysis

Univariate analysis was conducted using Pearson χ2 testing for categorical variables and Student’s t-test for continuous variables. Variables with a *p*-value < 0.1, along with sex, were included in a multivariate logistic regression model. Statistical significance for the multivariate regression was set at *p* < 0.05. All analyses were performed using IBM SPSS Statistics software Version 31 (Armonk, NY, USA).

## 3. Results

A total of 96 patients undergoing ACDF at 141 levels were identified as meeting the inclusion criteria. The implants used were sourced from five major manufacturers. Globus Medical accounted for the largest proportion with 45 implants, followed by Stryker (40), DePuy Synthes (25), Medtronic (15), and Caldera Medical (3). Regarding implant materials, the majority of interbody cages utilized were composed of polyetheretherketone (PEEK), totaling 96 cases. Titanium cages were used in 17 cases, and titanium-coated PEEK cages were used in 24 cases. In addition, allograft material was employed in 115 levels, either as a standalone graft or in conjunction with implantable devices.

### 3.1. Univariate Analysis

**Figure 1:**
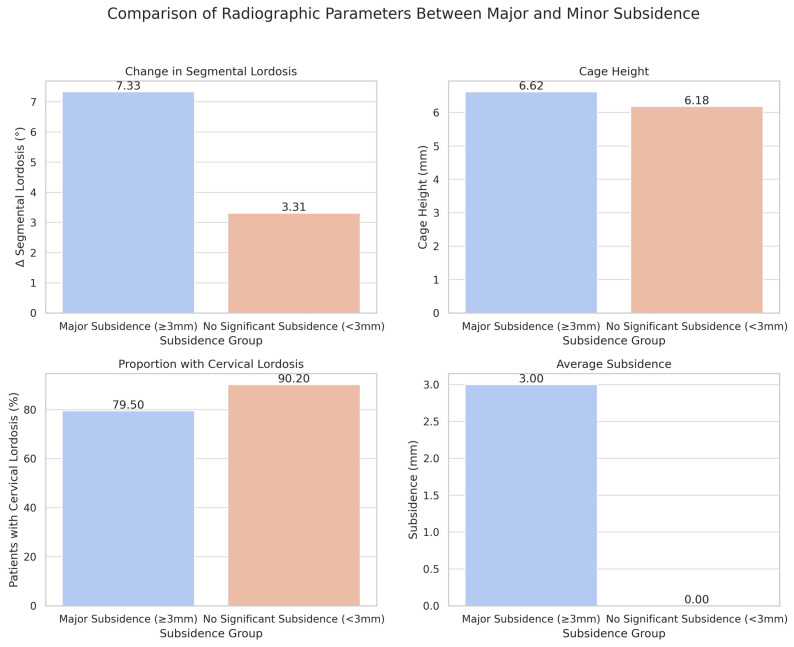
Comparison of Radiographic Parameters between Major and Minor Subsidence.

Among the 141 levels, 39 (28%) demonstrated significant subsidence of at least 3 mm, while 102 (72%) did not exhibit significant subsidence (Figure 1). Of the 96 patients, 31 (32.3%) had at least one level with significant subsidence, compared to 65 (67.7%) who did not experience significant subsidence. The average age of patients with significant subsidence was 52.44 years, while those without subsidence had an average age of 55.94 years (*p* = 0.074).

The proportion of male patients was slightly higher in the subsidence group (48.8%) compared to the non-subsidence group (40.2%), although this difference was not statistically significant (*p* = 0.360). There was a significantly higher incidence of standalone implants in levels that experienced subsidence (38.5%) compared to those without subsidence (16.7%) (*p* < 0.01). Additionally, the mean cage height for levels that subsided was larger (6.62 mm) compared to those that did not subside (6.18 mm) (*p* < 0.05).

The mean change in segmental lordosis from the postoperative period to the last follow-up also differed between the two groups. In the subsidence group, the change in segmental lordosis was +7.33°, compared to +3.31° in the non-subsidence group (*p* < 0.005). In the immediate postoperative period, 79.5% of levels that later subsided were associated with patients having cervical lordosis (defined as any angle less than zero degrees), while 90.2% of levels that did not subside were in patients with cervical lordosis (*p* = 0.088), as shown in Table 1.

### 3.2. Multivariate Analysis

A multivariate logistic regression was conducted, incorporating sex and the variables identified as significant in the univariate analysis: age, standalone cage, cage height, change in segmental lordosis, and postoperative cervical lordosis. The results revealed that standalone implants were an independent predictor of subsidence (OR 2.679; *p* < 0.05). Additionally, greater subsidence was positively associated with a greater loss of segmental lordosis (OR 1.089; *p* < 0.01).

However, the following variables were not found to be significant independent predictors of subsidence: age (OR 0.975; *p* = 0.206), male sex (OR 1.229; *p* = 0.626), cage height (OR 1.168; *p* = 0.411), and postoperative cervical lordosis (OR 0.820; *p* = 0.740).

## 4. Discussion

ACDF remains a cornerstone of treatment for degenerative cervical spine conditions, but the potential for subsidence continues to be a clinically relevant concern due to its association with alignment changes and fusion outcomes. The success of ACDF depends not only on achieving neural decompression but also on maintaining or restoring normal cervical alignment while promoting solid fusion. Subsidence represents a complex interplay of biological, mechanical, and technical factors that can compromise these objectives. ACDF with plate fixation is generally considered the benchmark for ensuring stable cervical interbody fusion in the treatment of degenerative cervical disorders. The use of an anterior plate fixation enhances cervical spine stability, preserves intervertebral height, and promotes higher fusion rates, thereby minimizing complications such as graft collapse and the loss of natural cervical curvature. The biomechanical advantages of plate fixation include load sharing, which reduces stress on the interbody graft, and the prevention of extrusion or migration of the interbody device. Additionally, plate fixation provides immediate stability that allows for early mobilization and potentially improved patient satisfaction. Despite its effectiveness, ACDF carries a notable risk of procedure-related morbidity, which led to the development of standalone spacers specifically designed for these procedures. The potential complications of plated constructs include dysphagia, which can be temporary or permanent, plate-related complications such as screw loosening or breakage, and the theoretical risk of stress shielding. These concerns have driven the development of standalone cages with features such as integrated fixation, expanded footprints, and surface treatments designed to enhance stability and integration. However, the impact of standalone spacers on operative characteristics and postoperative outcomes in ACDF cohorts, compared to cage and plate constructs, is an area of ongoing investigation in the literature. The debate over standalone versus plated constructs reflects the broader challenge in spine surgery of balancing simplicity and effectiveness. While standalone cages offer the advantages of reduced operative time, smaller incisions, and potentially lower morbidity, they may compromise stability and increase the risk of subsidence.

The notion that a standalone ACDF could be a safe and effective treatment for cervical degenerative disease has been a subject of debate in recent years, with several studies suggesting its viability. Proponents of standalone cages argue that modern cage designs with improved surface treatments, better anatomical fit, and integrated fixation features can provide adequate stability without the need for supplemental plating. However, critics contend that the absence of rigid fixation increases the risk of subsidence and may compromise fusion outcomes. Subsidence-related loss of segmental lordosis can lead to significant clinical consequences, including neural foraminal narrowing, radiculopathy, and adjacent segment degeneration. The biomechanical consequences of subsidence extend beyond the immediate operative level. Loss of segmental lordosis can alter the overall cervical alignment, potentially leading to compensatory changes at adjacent levels and increased risk of adjacent segment disease. Furthermore, subsidence can result in the loss of indirect decompression, potentially leading to recurrent symptoms.

The current investigation focuses on several important factors related to ACDF outcomes, with a particular focus on subsidence and its impact on segmental lordosis. One of the key findings is that standalone cages were an independent predictor of clinically significant subsidence (OR 2.679; *p* < 0.05) and that this subsidence resulted in a greater loss of segmental lordosis compared to patient’s preoperative scans (OR 1.089; *p* < 0.01). This finding has important clinical implications for surgical planning and patient counseling. The 2.7-fold increase in subsidence risk associated with standalone cages represents a clinically meaningful difference that should be considered in the decision-making process. Since restoring overall lordosis is one of the primary goals of an ACDF procedure, we found this to be a particularly noteworthy and significant finding. Although overall cervical lordosis did not differ significantly between the groups, the increased segmental collapse associated with subsidence could have biomechanical implications for adjacent segment kinematics and long-term fusion integrity.

The comparison of subsidence rates between standalone and plated cages has been explored previously in the literature, yielding inconsistent results. Wang et al. reported no significant difference in subsidence rates between the two implant types; however, their study presented a subsidence rate of less than 2%, which reflects a more conservative definition [12]. The variability in subsidence definitions across studies makes direct comparison challenging. Some studies define subsidence as any measurable change in disc height, while others use thresholds ranging from 1 mm to 5 mm. This inconsistency in definitions may explain the conflicting results in the literature. Other studies, often characterized by smaller sample sizes, may have lacked statistical power to detect meaningful differences [13,14,15]. In contrast, the present findings align with those of several studies and meta-analyses, including the work of Oliver et al. and Jin et al., which suggest that standalone cages are associated with higher rates of subsidence, even with follow-up durations extending up to two years [16,17,18,19,20]. The consistency of our findings with these larger studies provides confidence in the robustness of the results. The current study’s relatively large sample size and diverse patient population enhance the generalizability of the findings.

This study also found that subsidence correlates with a greater loss of segmental lordosis in the final postoperative follow-up imaging compared to the immediate postoperative period. Similar associations were observed in studies by Pinter et al. and Duey et al., which reported progressive segmental collapse [21,22]. However, other studies, including that by Ryu et al., found the prevalence of subsidence to be 48.1% but did not find significant clinical associations [23]. The discrepancy in clinical associations may be related to differences in patient populations, surgical techniques, or outcome measures. Additionally, the timing of the outcome assessment may influence the detection of clinical correlations, as some effects of subsidence may not become apparent until months or years after surgery.

Studies by Yang et al. and Igarashi et al. have demonstrated that subsidence primarily affects the anterior disc space, resulting in asymmetric collapse and forward vertebral tilt, which can accentuate kyphotic deformity. The asymmetric nature of subsidence has important implications for understanding its mechanism and clinical consequences. Anterior subsidence is more likely to be due to the relatively weaker anterior cortex of the vertebral body compared to the posterior cortex. This asymmetric collapse can lead to progressive kyphotic deformity, which may have long-term consequences for cervical alignment and adjacent segment health. Variability in findings regarding subsidence and lordosis loss may be attributed to differences in measurement methodologies [18,23]. Some studies, for example, assessed subsidence based on average anterior and posterior disc height changes, while others focused on maximum localized reduction. Additionally, variability in patient populations, surgical techniques, and follow-up durations contribute to inconsistent results. The methodology used in this study, which employed the average of the anterior and posterior disc height changes, was chosen to provide a more comprehensive assessment of subsidence that accounts for both symmetric and asymmetric collapse. This approach may provide a more accurate representation of the true magnitude of subsidence.

Standalone cages, identified as a significant predictor of subsidence in this study, may predispose patients to greater alignment loss compared to plated constructs due to the absence of supplemental fixation. This lack of additional support increases micromotion and stress concentration at the endplates, which can accelerate subsidence. The biomechanical rationale for increased subsidence with standalone cages is supported by finite element studies that demonstrate higher stress concentrations at the endplates in the absence of plate fixation. This increased stress can lead to endplate failure and progressive subsidence, particularly in patients with compromised bone quality. Although cage height was correlated with subsidence in univariate analysis, it was not an independent predictor in the multivariate model. However, excessive cage height may still contribute to endplate stress, particularly in patients with poor bone quality. The relationship between cage height and subsidence is complex and may depend on factors such as endplate preparation, bone quality, and cage design. While taller cages may restore disc height more effectively, they may also increase the risk of endplate fracture if the endplates are not adequately prepared or if the bone quality is poor. This has been noted by Pinter et al. and Igarashi et al., who found a correlation between cage height and subsidence but did not consider cage height an independent predictor [9,11,24]. These authors specifically examined cage height as a potential risk factor but concluded that while there was an observable relationship, the association was not strong enough to warrant classification as an independent predictor when controlling for other variables. In contrast, Yang et al. identified cage height as an independent predictor of subsidence, although their study utilized a lower threshold for cage height and did not stratify this variable, which may explain the discrepancies [8,25,26,27,28]. The methodological differences between these studies highlight the importance of standardized definitions and measurement protocols when evaluating cage height as a risk factor. Yang et al.’s use of a lower threshold (typically <6 mm vs. >8 mm in other studies) may have captured different patient populations and biomechanical scenarios, potentially accounting for the divergent conclusions regarding cage height’s predictive value.

The biomechanical rationale for cage height’s potential influence on subsidence relates to the distribution of axial loads across the endplate surface area. Taller cages may concentrate stress at the cage–endplate interface, particularly at the peripheral edges where the bone density is typically lower. Conversely, shorter cages may provide insufficient distraction to restore disc height and maintain cervical lordosis, leading to different patterns of settling over time. Patient-specific factors, such as bone mineral density and endplate integrity, are also critical [29]. Lower bone density is associated with higher subsidence rates, suggesting that preoperative assessments of bone health, such as DEXA scans, could help identify high-risk patients [30]. However, a significant limitation of the current study is the absence of systematic bone mineral density assessment via DEXA scanning in our patient cohort. This represents a critical gap in our analysis, as bone mineral density is widely recognized as one of the most important predictors of subsidence risk in spinal fusion procedures. The lack of objective bone health data may partially explain why some traditional risk factors failed to reach statistical significance in our multivariate model and limits our ability to provide comprehensive risk stratification for patients undergoing ACDF. The implementation of routine bone health screening protocols could enable surgeons to modify their surgical approach, select appropriate implant configurations, and initiate preventive measures before proceeding with ACDF procedures. Optimizing bone health through calcium and vitamin D supplementation or pharmacological interventions may reduce the risk of subsidence and its impact on alignment [31,32]. Recent evidence suggests that preoperative bone health optimization, including the use of bisphosphonates or other anti-resorptive agents in appropriate candidates, may significantly improve fusion rates and reduce subsidence risk. However, the timing and duration of such interventions require careful consideration to balance efficacy with potential complications. Future investigations should prioritize the incorporation of routine DEXA scanning and comprehensive bone health assessment as standard components of preoperative evaluation for ACDF patients. This would enable more accurate risk stratification and potentially improve patient selection for standalone versus plated constructs based on objective bone quality metrics.

Taken together, our findings support the importance of individualized implant selection and the potential benefit of using plated constructs in patients with known risk factors for subsidence, such as osteopenia or the need for multi-level intervention. This personalized approach to implant selection represents a shift from the traditional “one-size-fits-all” methodology toward a more nuanced understanding of patient-specific risk factors and their implications for surgical outcomes. The evidence suggests that plated constructs may provide superior resistance to subsidence through improved load distribution and enhanced stability, particularly in compromised bone environments.

This study, being a retrospective analysis, has several inherent limitations. First, loss to follow-up and selection bias may have introduced errors that could affect the results. The retrospective nature of this investigation inherently limits our ability to control for confounding variables and establish definitive causal relationships. Loss to follow-up rates of approximately 15–20% at long-term intervals may have created a systematic bias toward patients with either excellent outcomes (who do not require continued follow-up) or those with complications requiring ongoing care. Additionally, key factors such as smoking status, DEXA results, calcium levels, and other indicators of bone health were unavailable for analysis, which could have influenced subsidence and alignment outcomes. The study was also conducted at a large institution with multiple surgeons, which may have introduced variability in surgical techniques and implant selection, further impacting the results. Inter-surgeon variability in technique, including differences in endplate preparation, implant sizing, and positioning, could have influenced outcomes in ways that our analysis was unable to capture. While this multi-surgeon approach enhances the generalizability of our findings, it also introduces potential confounding factors that may have masked or amplified certain relationships. The use of different implant models could have contributed to these variations. The heterogeneity in implant design, including variations in surface texturing, material composition, and geometric configuration, may have created subgroups with different subsidence characteristics that were not adequately captured in our analysis. Finally, the absence of long-term follow-up data for many patients limits the ability to predict long-term radiological outcomes and the progression of subsidence over time. This limitation is particularly significant given that subsidence patterns may evolve over months to years following surgery, and our ability to detect late-occurring complications or progressive alignment changes is therefore constrained.

## 5. Conclusions

This large cohort study demonstrates that standalone implants are an independent predictor of subsidence in ACDF, with subsidence being associated with a significant loss of segmental lordosis. The additional stability provided by anterior plating may be beneficial in preventing implant settling, particularly in high-risk patients. While subsidence did not significantly affect overall cervical alignment, suggesting effective compensatory mechanisms, the long-term clinical consequences remain unclear. Future prospective studies incorporating objective bone health assessments and extended follow-up are needed to optimize implant selection and predict subsidence risk in ACDF patients.

## Figures and Tables

**Table 1:** Comparison of demographic, surgical, and radiographic characteristics between operative levels with and without subsidence.

Variable	Subsidence (*n* = 39 Levels)	No Subsidence (*n* = 102 Levels)	*p*-Value
Proportion of Subsided Levels	28%	72%	–
Proportion of Patients With ≥1 Subsided Level	32.3% (31/96)	67.7% (65/96)	–
Mean Age (years)	52.44 ± X	55.94 ± X	0.074
Male Sex (%)	48.8%	40.2%	0.360
Standalone Implant Use (%)	38.5%	16.7%	<0.01
Mean Cage Height (mm)	6.62	6.18	<0.05
Mean Change in Segmental Lordosis (°)	+7.33	+3.31	<0.005
Post-op Cervical Lordosis Present (%)	79.5%	90.2%	0.088

## Data Availability

The datasets generated and/or analyzed during the current study are available from the corresponding author upon reasonable request.

## Abbreviations

The following abbreviations are used in this manuscript:

ACDF	Anterior Cervical Discectomy and Fusion
PEEK	Polyetheretherketone
BMI	Body Mass Index
SVA	Sagittal Vertical Axis
